# Protocol: Personality assessment as a support for referral and case-work in treatment for substance use disorders (PASRC-study)

**DOI:** 10.1186/1471-244X-8-30

**Published:** 2008-04-25

**Authors:** Morten Hesse, Mads K Pedersen

**Affiliations:** 1 University of Aarhus, Centre for Alcohol and Drug Research, Købmagergade 26E, 1150 Copenhagen C, Denmark

## Abstract

**Background:**

Assessment of co-morbid personality disorders in substance use disorders may lead to important insights concerning individual patients. However, little is known about the potential value of routine personality disorder assessment in a clinical context.

**Methods:**

Patients are adults with past-year substance dependence seeking treatment at a centralized intake unit for substance abusers in the City of Copenhagen. A randomized controlled trial of assessment of personality disorders and individual feedback vs. a general life situation interview. Patients are followed at 3 and 6 months post-treatment

**Discussion:**

If routine personality assessment improves outcomes of substance abuse treatment, the clinical implication is to increase the use of personality disorder assessment in substance abuse treatment settings.

**Trial registration:**

Current controlled trials ISRCTN39851689

## Background

Substance use disorders and personality disorders often co-occur [1,2]. Patients with personality disorders are commonly seen in treatment programs for substance abuse, consume a disproportionate amount of staff time, and are more likely to drop out from substance abuse treatment interventions [3,4].

At the same time, therapists and other professionals tend to react negatively to patients with personality disorders at an emotional level [5], this is especially the case with cluster B (dramatic/erratic) personality disorders [6].

Even if patients with personality disorders benefit from treatment, they often remain more symptomatic than patients without personality disorders, and remain at a lower level of functioning [7,8].

Therefore, treatment that meets the needs of patients with substance abuse and personality disorders is needed. Recently, some studies have shown that integrated treatment for personality disorders and substance abuse may be superior to treatment that focuses solely on substance abuse treatment [9-11].

This randomized experimental study is designed to assess whether systematic assessment of personality disorders improves outcomes, vs. assessment of Axis I disorders alone.

The objective of this study is to estimate the effect of routine assessment of co-morbid axis II disorders in a centralized intake unit for substance use disorders.

## Method

### Design

The study is a randomized experimental trial comparing assessment of Axis I disorders alone with assessment of both Axis I disorders and Axis II disorders. For both treatment conditions, patients are given feedback about the results of assessment and offered the opportunity to have their key-worker receive the same feedback.

### Participants/setting

Participants are adults seeking treatment for a substance use related disorder at the Central Intake Unit (CIU) in Copenhagen, Denmark (Købmagergade 26E, St., 1150 Copenhagen C).

### Referral and recruitment

Patients are recruited by caseworkers. The management of the intake unit and the management of the centre have instructed all caseworkers to ask all new referrals or those referred for a change in treatment to participate in the study.

Caseworkers inform patients that the study concerns assessment of psychological problems, behaviour and other disorders, and that patients will be randomly assigned to one of two types of interviews. The primary interviewer later gives detailed information about the study procedure.

### Inclusion criteria

To be eligible for the study, patients must satisfy the following criteria:

• Be at least 18 years of age.

• Not currently be psychotic or have a known diagnosis of schizophrenia excluding patients from treatment at the intake unit.

• Not currently be involved in an ongoing treatment for a drug or alcohol problem. Patients who are currently involved in treatment for alcohol or drug abuse will, however, not be referred to the CIU.

• Have past-year substance dependence, as indicated by a score on the SDS of 3 or more for either alcohol or drugs.

• Speak Danish or English fluently.

• Give informed consent.

The assessment and feedback takes place in the first three weeks of treatment.

**Figure 1:**
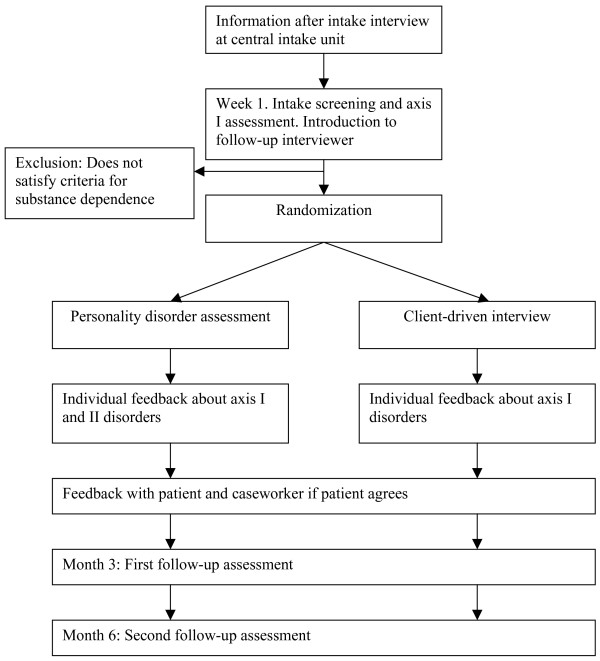
Flowchart of the process from information to follow-up.

**Figure 2:**
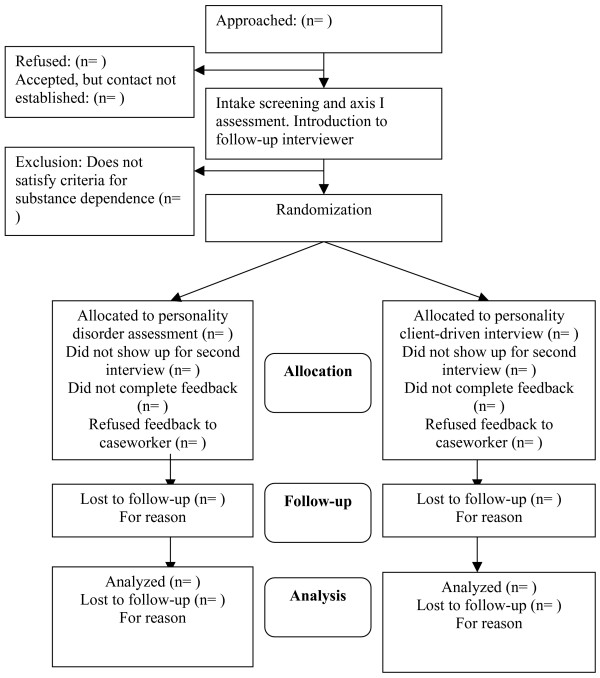
Flowchart of participants.

Table 1 gives an overview of the interviews and questionnaires conducted at the different assessment times. Figure 1 represents the research procedure schematically. Figure 2 contains the expected participant flow.

**Table 1:** Instruments at different assessment moments

	Baseline	Follow-up	Follow-up
Interview:			
SAPAS [22, 23]	*	*	*
ASRS [14]	*	*	*
K6 [32]	*	*	*
OTI [15]	*	*	*
SDS – alcohol [21]	*	*	*
SDS – drugs [21]	*	*	*
WAI [29]		*	
Ratings of the feedback procedure – VAS		*	
Clinician rated:			
CGI-externalizing [23]		*	*
CGI-substance use [23]		*	*
GAF [23]		*	*
WAI [29]		*	

**Notes**: *SAPAS*: Standardised Assessment of Personality – Abbreviated Scale. *ASRS*: Adult ADHD Self-Report Scale. *K6*: Kessler 6. *OTI*: Opiate Treatment Index. *SDS*: Severity of Dependence Scale. *WAI*: Working Alliance Inventory. *VAS*: Visual analogue scales. *CGI*: Clinical Global Impression. *GAF*: Global Assessment of Functioning.

### Outcome criteria

The main outcome criterion will be the following: Improvement in at least one of the following areas, described as a 30% decline in score, with no concurrent deterioration in any area: global functioning, as measured by the Work and Social Adjustment Scale [12]; psychiatric symptoms, as measured by the Kessler 6+ [13,14]; substance use, as measured by the Opiate Treatment Index drug use indicator [15]; treatment engagement, as measured by treatment staff, using the treatment engagement scale from DATOS [16].

Secondary outcome criteria will be

• Retention in treatment, defined as being retained in the same treatment that patients were first referred to after their contact with the intake unit at 3 and 6 months follow-up, or having completed that treatment as planned.

• Understanding of one's own personality and the impact that personality has on others, as rated by an independent interviewer [17].

• Readiness to change dysfunctional behaviour, work with emotional problems, change illicit drug use, and change alcohol use, as measured by brief 4-item questions, adapted from the smoking cessation literature [18].

Outcome criteria will be assessed at 3 and 6 months follow-up.

### Intake procedure

At intake to the CIU, patients undergo a routine intake interview with a key-worker that includes the Addiction Severity Index [19]. After the completion of the intake interview, patients are informed by the caseworker of the fact that a study of psychopathology is ongoing at the unit. If the patient agrees to receive further information, a researcher is contacted, which immediately comes to the office of the key-worker to meet the patient and schedules an intake interview.

### Baseline interview

At the baseline interview, the interviewer informs the patient of the study, and explains that the study focuses of psychological and behavioural problems that people with substance abuse regularly encounter. He then explains that there are two different interviews, and that patients will be randomly assigned to one of two types. If asked, he will explain that he does not yet know what kind of interview he will conduct the second time they meet, but he will give a further explanation at their second appointment. If the patient gives consent to participate in the study, a brief structured assessment of axis I disorders is conducted. The assessment is kept brief, in order to reduce assessment reactivity, i.e., the effects that assessment can have on treatment outcomes [20].

The instruments used are therefore chosen to be brief, but have strong indications of validity:

• Anxiety/depression is assessed with The Kessler 6 interview [14].

• Attention Deficit/Hyperactivity Disorder is assessed with the Adult ADHD Self-Report Scale [14].

• Illicit substance dependence and alcohol dependence are both assessed by means of the Opiate Treatment Index substance use items [15].

• Severity of alcohol and illicit drug dependence will be assessed by the Severity of Dependence Scale [SDS] [21].

• Personality disorder severity is assessed with the Structured Assessment of Personality – Abbreviated Scale [SAPAS] [22,23].

If the patient does not speak Danish or English well, does not screen positive for substance dependence as indicated by an SDS score of 3 or more for either alcohol or drugs, a feedback session is scheduled, and the patient is informed that based on his data, we wish to give him individual feedback on the next session.

If the patient satisfies inclusion criteria (speaks Danish or English; SDS > 3; gives informed consent), a second interview is scheduled. The interviewer will also schedule a meeting with the researcher conducting follow-up interviews.

The patient is then randomized to either experimental or control conditions.

### Second interview – experimental condition

If the patient is randomized to the experimental condition, the second interview will be an assessment of axis II disorders. The patient will first receive a brief description of what personality disorders is, and be given a brief description of personality disorders as inflexible, maladaptive patterns of behaviour that cause significant problems or distress for themselves or others.

The interview will consist of the following elements:

• The Alcohol Use Disorder and Associated Disorders Interview Schedule (AUDADIS) section for avoidant, dependent, obsessive-compulsive, paranoid, schizoid and histrionic personality disorder [24]. Added items taken from the Parker Personality inventory will be taken in to reflect schizotypal personality disorder and narcissistic personality disorder [25].

• The NPI-16 will be added as a measure of narcissism [26].

• The Psychiatric Research Interview for Substance and Mental Disorders (PRISM) [27,28] will be used to assess borderline and antisocial personality disorder.

Following this, patients will be asked if they have any questions, and thereafter the interviewer will proceed with the interview. The interview will contain the following elements.

The interviewer will be trained through role-plays in administering the interview package. For the PRISM, taped interviews will be co-rated to assess the inter-rater reliability of coding.

### Second interview: control condition

The control condition will contain an interview of up to one hour, where the patient chooses focus based on a list of items (substance use problems; family; friends; work/education; etc). The interview is client-driven and follows an ethnographic approach.

These interviews are taped and used as the basis for a condensed summary in the feedback

### Randomization and blinding

Randomization will be conducted by means of a predefined list of random numbers, stratified by predefined characteristics, which will not be disclosed at this point to reduce risk of breaking of blind. Randomization is executed according to a list, the allocation sequence of which was computer-generated by one of the researchers (MH). The interviewer will be blinded to randomization at the baseline interview.

The follow-up interviewers will be introduced to the patients at the baseline interview, to assure blinded follow-up assessments.

### Withdrawal

A participant can withdraw from the trial at any point, although information already given to caseworkers cannot be withdrawn. Participants who withdraw from the trial treatment will not be asked to attend the follow-up appointments, and will be deleted from all files.

### Feedback and psychoeducation procedure

Patients will receive individual feedback first. The feedback is inspired by psychoeducation procedures [17]. For each diagnosis for which a positive result is found, patients are first prompted about their knowledge of the disorder, and then given their test results. Test results will involve diagnoses and possible treatment options and implications (e.g., psychotherapy and/or medication for anxiety/depression; skills training and/or medication for attention deficit/hyperactivity disorder). For personality disorders, treatment implications, resources and relevant treatment options are summarized in Table 2.

**Table 2:** Treatment implications, personal resources, and relevant treatment options for each of the ten personality disorders

**Personality disorder**	**Implications**	**Resources**	**Treatment options**
Paranoid	Problems dealing with high expressed emotion; needs time to build trust; needs great patience; problems with groups, especially confrontative groups.	Careful, able to cope in realistic danger; protects own privacy	Counselling; inpatient treatment in small wards with great flexibility; not exploratory psychotherapy [10]
Schizoid	Problems dealing with high expressed emotion; needs great patience; does not benefit from requests for participation in social activities.	Able to deal with being alone;	Counselling; possibly inpatient treatment in small wards with great flexibility [10]
Schizotypal	Problems dealing with high expressed emotion; needs great patience; problems with groups; needs time to build trust.	Creative, independent thinking	Counselling; possibly inpatient treatment in small wards with great flexibility [10]; antipsychotic medication
Antisocial	Impulse actions; "plays the game"; needs straight talk from counsellor or case worker; transgresses boundaries in treatment;	Great potential for action under many circumstances	Therapeutic community or similar treatment [33]; regular addictions treatment; cognitive-behavioural interventions, or similar
Borderline	Impulse actions; transgresses boundaries in treatment; needs to learn to cope with emotion; chaotic relationships to therapists	Sensitive and able to experience emotions	Psychotherapeutic treatment; antidepressants; antipsychotics; inpatient treatment; long-term involvement
Histrionic	Flirts and appears shallow and superficial to others; has difficulties focusing on own situation and issues	Charming and outgoing	Psychotherapeutic treatment; counselling; inpatient treatment; cognitive-behavioural interventions
Narcissistic	Appears grandiose and arrogant; makes it difficult for staff members to intervene ("scares" away all criticism)	Has ability to feel pride	Inpatient treatment for drug misuse; self-change program, cognitive-behavioural interventions
Avoidant	Difficulty getting out with new people; stays in "safe zones", and has difficulty trying out new treatment options or seeking social support or employment	Self-protective; sometimes able to stay out of trouble by keeping away	Psychotherapeutic treatment; antidepressants; inpatient or outpatient treatment; individual counselling and case management
Dependent	At high risk of abusive relationships	Good ability to form working relationships; good compliance	Psychotherapeutic treatment; antidepressants; inpatient or outpatient treatment; individual counselling
Obsessive-compulsive	Difficulties concluding in counselling or therapy settings; attempts to control counsellor, and other professionals	Sticks to goals	Psychoeducation;

### Follow-up procedure

Trained interviewers will make contact with study participants before randomization, but after intake interview. Interviewers will be blinded to randomization status. The follow-up interview will contain the same elements as the baseline interview (i.e., the K6, the ASRS, the WSAS, the OTI, and the SDS). Also, patients will be asked to rate the value of the feedback procedure on visual analogue scales representing the skills of the interviewer (on a line ranging from "extremely unskilled" to "extremely skilled"), his knowledge (from very little knowledge, to highly knowledgeable), his degree of interest in the patient, his understanding of the patient, the value of the feedback for the treatment, the value of the treatment for the patient, how much thought the patient has given the feedback, and how much work he has done in regard to the issues discussed in the feedback.

Finally, the patient is asked to rate his alliance with the caseworker using the Working alliance inventory, patient version [29].

At each interview point, the caseworker is asked to rate the patient using the clinical global impression scale with anchor points for alcohol and drug abuse [23], externalizing behaviour [23], and GAF [23], and to complete the Working Alliance Inventory, clinician version [29].

### Data handling and record keeping

Patient information will only be accessible to the research team. All data will be link-anonymised so that no patient identifying information will be kept with raw data. All files will be kept with the local research teams in a locked and secure cabinet. Electronic data will be stored on a central computer at the research centre.

A personal feedback letter will be given to the patient, in which the patient appears only with his or her first letter. If the patient agrees that the information in the personal feedback should be shared with the caseworker, then the same letter will be given to the caseworker. Raw data will, however, only be kept and stored by the Centre for Alcohol and Drug Research.

### Outcome evaluation

A single measure of outcome will be used as the primary outcome measure: The proportion of areas in which a 30% reduction occurred at each follow-up wave (K6, WSAS, and OTI substance use). This measure can theoretically range from -3 (deterioration in all areas) to +3 (improvement in all areas.

The data will be analyzed with mixed effects ordinal regression, using baseline values of the three outcome measures as covariates [30].

### Risks and anticipated benefits for trial participants and society, including how the benefits justify the risks

Patients with substance use disorders and co-morbid personality disorder experience a number of serious problems. Patients with co-morbid personality disorder tend to remain symptomatic long after treatment [7,8], and patients with some co-morbid personality disorders tend to commit a substantial amount of the crime that substance abusers commit [31].

If the findings of this study show that personality assessment can significantly improve the functioning of patients with substance use disorders, this can have significant impacts on both the quality of life of patients, and on society.

The main risk of this study is that patients experience a worsening of psychiatric symptoms or substance use as a reaction to receiving a feedback concerning their own personal functioning. There is some indication that symptoms may fail to improve with focus on personality, even if this focus improves substance use and therapeutic alliance [9]. We will monitor psychiatric symptoms every 3 months during the study, and patients have access to caseworker, psychiatrist and psychologist at the treatment centre.

### Ethical approval

Institutional Review Boards in Denmark do not approve or disapprove of trials or other studies of psychosocial interventions. The medical director of social medicine in the City of Copenhagen, M.D. Peter Ege, read and approved the protocol in Danish. Patients will be given full information about the nature of the study, and be asked to give informed consent. Patients who refuse participation in the study will be given full access to all regular treatment services in the organisation, similar to the control group.

Ethically, the issues related to this study concerns the consequences of receiving serious diagnoses for which there is no clear and simple treatment, in particular personality disorders. However, although there is no simple treatment, patients are given suggestions and support, and have access to the support of the treatment centres

When patients in this trial are assessed, they also receive feedback and advice about how to handle the issues involved, and their caseworkers are given feedback and advice on how to best support patients.

## Discussion

This paper describes the study protocol of a randomized controlled trial concerning personality disorder assessment and feedback versus an open interview. The aim of this study is to assess the potential value of personality disorder assessment for referral and casework in a real-life setting. The study design described above has specific strengths and limitations.

The study has a single-blind design, where patients are unaware if their treatment is designated as an "experimental" or "control" treatment. Follow-up interviewers are blinded to allocation, and we have procedures to reduce the risk that follow-up interviewers are made aware of subjects' status (i.e., follow-up interviewers make contact with patients before randomization is known; patients are told not to disclose details about the interview to the follow-up interviewers).

The study uses an attention placebo design, which means that the effects of time and attention are controlled for. Research has shown strong assessment reactivity in substance use outcome [20], meaning that patients who are exposed to assessment show improvement only for that reason. The amount of contact is controlled for in this trial. This means that if significant effects are found, they give strong support to the efficacy of personality disorder assessment.

The setting in which the study takes place is a community service. This means that although training and supervision is provided for caseworkers, the caseworkers are not primarily working with the research project. While this may reduce the potential efficacy of the intervention, it means that the study is in a sense between an efficacy trial and an effectiveness study.

## Competing interests

The first author of the article, MH, occasionally does paid training in understanding and assessing personality disorders.

## Authors' contributions

MH originally conceived of the study, and wrote the application to the Health Insurance Fund. MH drafted the manuscript, but both authors reviewed it and MKP added substantial practical details and points to the manuscript.

## Pre-publication history

The pre-publication history for this paper can be accessed here:


